# Editorial: *Candida auris* - understanding the new superbug

**DOI:** 10.3389/fcimb.2023.1241623

**Published:** 2023-07-05

**Authors:** Sukalyani Banik

**Affiliations:** Center for Emerging Pathogens, Department of Medicine, New Jersey Medical School, Rutgers University, Newark, NJ, United States

**Keywords:** *Candida auris*, identification, anti-fungal, multi-drug, resistance, virulence

*Candida auris* is very much in the news these days. Since it was first reported over a decade ago, *C. auris* has been reported in more than 30 countries and linked with many nosocomial outbreaks in healthcare facilities worldwide. Its resistance to multi-antifungals, misidentification by standard laboratory methods and long-term survival within hospital and long-term care settings have prompted the US Centers for Disease Control and Prevention to classify *C. auris* as an “Urgent Threat” to public health. Earlier this year, *C. auris* was listed as one of the four “critical” threats on the WHO’s first-ever list of fungal priority pathogens. This Research Topic incorporates recent studies on different approaches to identify *C. auris*, biological factors contributing to its virulence and mechanisms of its anti-fungal resistance.

Available diagnostic tests for *C. auris* require a few hours to a few days for identification, demand higher technical skills using complex methods and often lead to misidentification for other infections. Arastehfar et al. developed a tetraplex end-point PCR that can detect and differentiate *C. auris* from its phylogenetically closely related species *C. haemulonii*, *C. duobushaemulonii* and *C. pseudohaemulonii*. They also highlighted the importance of including these four species in their multiplex panel as they all are highly multidrug-resistant pathogens and often misdiagnosed by routinely used identification systems. Their assay was highly specific and validated in both prospective and retrospective cohorts. Through these cohorts, they reported the first clinical isolates of *C. haemulonii* and *C. pseudohaemulonii* in Iran and China respectively. They were the first to validate their assay in an animal model. Pezzotti et al. applied the Raman spectroscopic method not only to differentiate *C. albicans* from *C. auris* but also to distinguish clade II and clade III of *C. auris*. Besides accurate identification, their Raman analyses revealed some fundamental differences related to the polysaccharide structure of the cell membrane, the ergosterol levels in the plasma membrane, virulence and drug resistance features of different species of *Candida*. A year later Pezzotti et al. showed that Raman spectroscopic method was also a valuable tool to understand the susceptibility and stress response of different clades of *C. auris* to different antifungal drugs. Their research also highlighted how different *C. auris* clades reacted to different antifungal drug treatments and discovered the regulatory mechanisms to compensate for any structural changes.

Since the start of the COVID-19 pandemic, there have been increasing reports of *C. auris* outbreaks occurring in hospitals and acute care facilities. Patients hospitalized for severe COVID-19 are at risk of contracting nosocomial infections including candidemia or invasive candidiasis due to their weakened immune system. Secondary hemophagocytic lymphohistiocytosis (sHLH) is a life-threatening hyperinflammatory event triggered by viral infections including SARS-CoV-2 which leads to multiorgan failure. Gautam et al. reported a case where a patient developed a disseminated infection with *C. auris* after developing sHLH from SARS-CoV-2 infection.

*C. auris* has been responsible for many outbreaks of multi-drug resistance infections in healthcare settings around the world and thus has become a major health concern. Despite expressing fewer virulence factors than *C. albicans*, *C. auris* has the ability to spread more rapidly within and between long-term care facilities. Rossato and Colombo summarized all biological factors contributing to the pathogenicity of *C. auris*. Although the role of different genes in the pathogenicity and virulence of *C. auris* is still unknown, a significant portion of its genome encodes transporter genes such as ABC (ATP Binding Cassette) and MFS (Major Superfamily Facilitator) transporters along with oligopeptide and iron transporters. Wasi et al. analyzed the entire repertoire of ABC transporters of *C. auris* type strain CBS 10913T and identified 28 putative ABC proteins. They also confirmed the presence of 20 TMD (transmembrane domain) proteins and their involvement in multidrug resistance in *C. auris*.

*C. auris* is an emerging multi-drug resistant fungal pathogen. It has become a major threat to public health as the number of drug-resistant cases has been increasing. This Research Topic highlighted new insights into *C. auris* genomes to combat this deadly pathogen and rapid identification for effective control measurements. We would like to thank all authors for their contribution to this Research Topic.

## Author contributions

The author confirms being the sole contributor of this work and has approved it for publication.

